# Plant-specific features of respiratory supercomplex I + III_2_ from *Vigna radiata*

**DOI:** 10.1038/s41477-022-01306-8

**Published:** 2022-12-29

**Authors:** M. Maldonado, Z. Fan, K. M. Abe, J. A. Letts

**Affiliations:** 1 Department of Molecular and Cellular Biology, University of California, Davis, CA USA; 2 Present Address: Department of Plant Biology, University of California, Davis, CA USA

**Keywords:** Cryoelectron microscopy, Biochemical assays, Plant sciences, Plant molecular biology

## Abstract

The last steps of cellular respiration—an essential metabolic process in plants—are carried out by mitochondrial oxidative phosphorylation. This process involves a chain of multi-subunit membrane protein complexes (complexes I–V) that form higher-order assemblies called supercomplexes. Although supercomplexes are the most physiologically relevant form of the oxidative phosphorylation complexes, their functions and structures remain mostly unknown. Here we present the cryogenic electron microscopy structure of the supercomplex I + III_2_ from *Vigna radiata* (mung bean). The structure contains the full subunit complement of complex I, including a newly assigned, plant-specific subunit. It also shows differences in the mitochondrial processing peptidase domain of complex III_2_ relative to a previously determined supercomplex with complex IV. The supercomplex interface, while reminiscent of that in other organisms, is plant specific, with a major interface involving complex III_2_’s mitochondrial processing peptidase domain and no participation of complex I’s bridge domain. The complex I structure suggests that the bridge domain sets the angle between the enzyme’s two arms, limiting large-scale conformational changes. Moreover, complex I’s catalytic loops and its response in active-to-deactive assays suggest that, in *V. radiata*, the resting complex adopts a non-canonical state and can sample deactive- or open-like conformations even in the presence of substrate. This study widens our understanding of the possible conformations and behaviour of complex I and supercomplex I + III_2_. Further studies of complex I and its supercomplexes in diverse organisms are needed to determine the universal and clade-specific mechanisms of respiration.

## Abstract (web summary)

Cryogenic electron microscopy structure of respiratory supercomplex I + III_2_ from mung bean reveals interfaces, a new complex I subunit and differences in complex III_2_ versus other supercomplexes. Resting complex I adopts an intermediate conformational state and a non-canonical active/deactive response.

## Main

Cellular respiration is an essential metabolic process in plants. The last steps of respiration are carried out by oxidative phosphorylation (OXPHOS) in the inner mitochondrial membrane (IMM). In its canonical form, OXPHOS involves four multi-subunit protein complexes of the electron transport chain (complexes I–IV, CI–CIV) as well as the mitochondrial ATP synthase (complex V). Complexes I–IV transfer electrons from NADH or succinate to molecular oxygen through the electron carriers quinone and cytochrome *c* and establish an electrochemical proton gradient across the IMM. This proton gradient is then dissipated by complex V, producing ATP^1^. OXPHOS complexes can exist independently, but more frequently assemble into stoichiometric higher-order assemblies called supercomplexes, whose functions have remained unclear^2–7^. In plants, between 50% and 90% of CI has been found associated with CIII_2_ (SC I + III_2_) after detergent extraction from the IMM and native-gel electrophoresis, which probably underestimates the extent of supercomplex associations in vivo^8^. Therefore, to fully understand the physiological functions of CI—the main entry point of electrons into OXPHOS—we must study it in the context of its supercomplexes. The biochemical and structural analysis of SC I + III_2_ is a crucial step towards understanding respiratory supercomplexes in plants.

Recently, multiple cryogenic electron microscopy (cryoEM) structures have been obtained for plant CI or CI fragments^9–11^. Additionally, structures for SC I + III_2_ from *Ovis aries* (ovine) and *Tetrahymena thermophila* are available^12,13^. While these structures aid our understanding of plant SC I + III_2_, they are not sufficient. For instance, all structures of plant CI are incomplete, missing subunit NDUA11 as well as density for transmembrane helices (TMHs) in core subunits Nad5 and Nad6. Additionally, several densities have remained unassigned, including that for a putative new subunit whose identity could not be determined^9–11^. Moreover, sequences involved in SC I + III_2_ formation in *O. aries* and *T. thermophila* (for example, from NDUB4 and NDUB9) are missing in the plant homologues, and there are substantial differences in the SC I + III_2_ interfaces between ovine and *T. thermophila* SC I + III_2_. Therefore, these structures cannot directly be used to make predictions about the plant SC interface(s). Furthermore, although reconstructions of plant SC I + III_2_ are available from subtomogram averages from *Asparagus officinalis*^7^ and negative-stain electron microscopy from *Arabidopsis thaliana*^14^, their resolutions are too low to make detailed assessments.

In this Article, we present a high-resolution structure of SC I + III_2_ from *Vigna radiata* (mung bean) from a biochemically active preparation. This structure contains the full complement of subunits for plant CI including NDUA11 (B14.7), previously missing fragments from Nad5 and Nad6, and a newly assigned CI subunit (NDUP9). Moreover, CIII_2_ contains a different isoform of mitochondrial processing peptidase (MPP)-α subunit from the one observed in isolated CIII_2_ and SC III_2_ + IV (ref. 15). CI and CIII_2_ interact at three sites that do not involve the bridge domain of CI. Contrary to a previous hypothesis from *A. thaliana*’s CI (ref. 11), our analysis suggests that the opening of the complex is more likely due to sample degradation than to a regulatory process. Moreover, an examination of CI’s large-scale structure, catalytic loops and active-to-deactive (A/D) response suggests that *V. radiata* CI’s resting conformation is an intermediate, non-canonical state and that CI samples similar states in both the absence and presence of substrate.

## Structure of *V. radiata* SC I + III_2_ shows complete CI and differences in CIII_2_

**Fig. 1:**
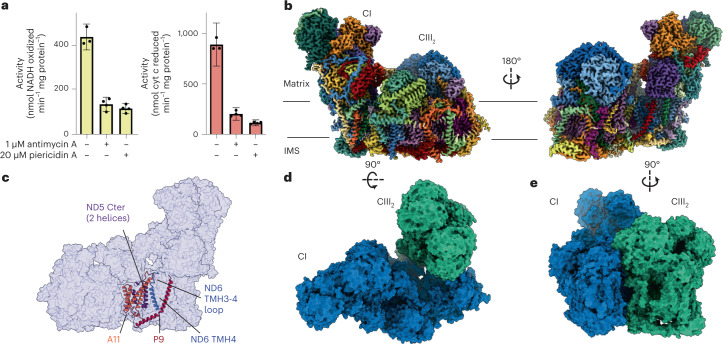
Structure of *V. radiata*’s SC I + III_2_. **a**, NADH-cyt *c* oxidoreductase activity of amphipol-stabilized isolated SC I + III_2_ in the absence or presence of CI or CIII_2_ inhibitors (20 μM piericidin A and 1 μM antimycin A, respectively). Values are averages of three or four independent measurements from a single purified sample of SC I + III_2_; error bars display the coefficient of variance calculated as the sum of the coefficients of variation of each experimentally determined value (path length, extinction coefficient and activity) multiplied by the average. **b**, CryoEM density map of SC I + III_2_ coloured by subunit. The approximate locations of the mitochondrial matrix and intermembrane space (IMS) are shown with black lines. **c**, Atomic model improvement versus previously available structures of plant CI. Improved or new subunits shown in coloured cartoons over CI semi-transparent surface. Cter, C-terminus. **d**,**e**, SC I + III_2_ shown from the matrix (**d**) or the plane of the membrane (**e**). Complex I (CI) in blue surface, complex III_2_ (CIII_2_) in green surface.

**Extended Data Fig. 1:**
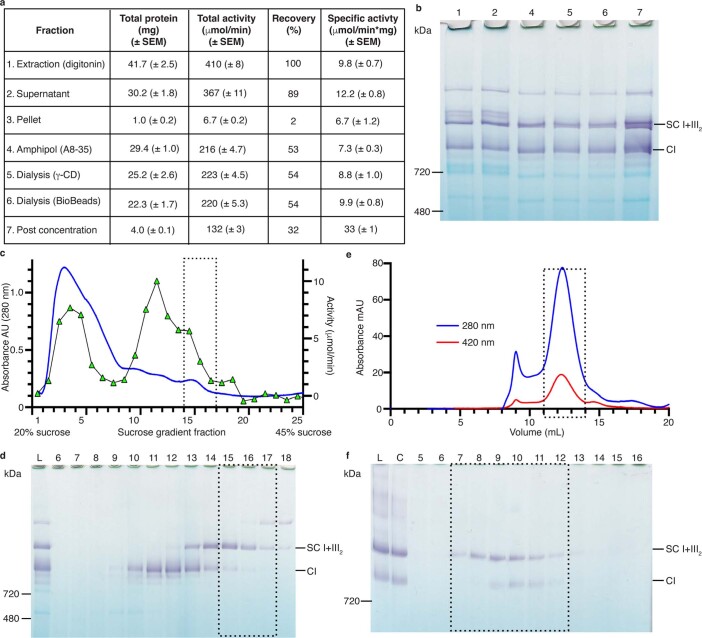
Biochemical purification of *V. radiata* SC I + III2. (**a**) Purification table detailing protein concentration (mg/ml), total activity (μmol/min), specific activity (μmol/(min*mg) and recovery (%, normalized to extracted protein) for each main purification step. (**b**) Complex I (CI) in-gel activity assay of fractions detailed in (a) using blue-native PAGE. Purple bands indicate CI activity. (**c-d**) Sucrose gradient fractionation of post-concentration fraction (7 in panel a) after ultracentrifugation. (**c**) Sucrose gradient chromatogram (blue line, absorbance at 280 nm) and total activity (μmol/min) (green triangles, determined with spectroscopic ferricyanide assay) for each sucrose-gradient fraction. Dashed box indicates pooled fractions for size exclusion column (SEC). (**d**) CI in-gel activity assay of selected fractions from (c). (**e-f**) SEC purification of fractions pooled from (c-d). (**e**) Chromatogram at 280 nm and 420 nm. (**f**) CI in-gel activity assay of fractions collected from (e). Fractions corresponding to SC I + III_2_ marked with dashed box. C, post-SEC concentrated sample; L, load. These results are representative of 26 SC I + III_2_ preparations. Source data

We isolated mitochondria from etiolated mung bean hypocotyls. We then extracted protein complexes from the mitochondrial membranes using the gentle detergent digitonin and stabilized them using amphipathic polymers (Extended Data Fig. 1a,b). Using a sucrose gradient, we partially purified respiratory complexes and supercomplexes (Extended Data Fig. 1c,d). We pooled, buffer-exchanged and concentrated the fractions containing SC I + III_2_ (Extended Data Fig. 1c,d) and used this partially purified sample to blot cryoEM grids, as previously described^9,13,15^. We further purified SC I + III_2_ using size-exclusion chromatography (SEC) (Extended Data Fig. 1e,f). The final biochemical sample showed the expected NADH-cyt *c* oxidoreductase activity, which was inhibited by CI and CIII_2_ inhibitors piericidin A and antimycin A, respectively (Fig. 1a).

**Extended Data Fig. 2:**
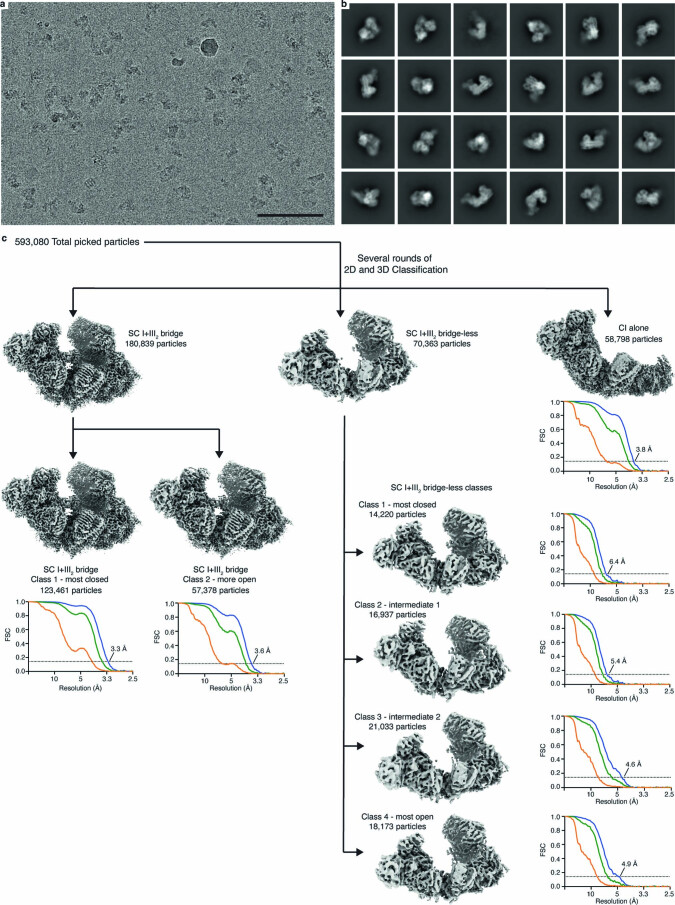
Micrograph and initial processing. (**a**) Representative micrograph of 21,815 collected. Scale bar is 100 nm. (**b**) Representative 2D classes of SC I + III_2_ particles. (**c**) Initial 3D classification and refinement pipeline. Fourier shell correlation (FSC) curves for the individual class refinements are shown for no (orange), loose (green) and tight (blue) masking. The resolution at which the tight masked FSC crosses the 0.143 gold-standard limit (dashed line) is indicated.

**Extended Data Fig. 3:**
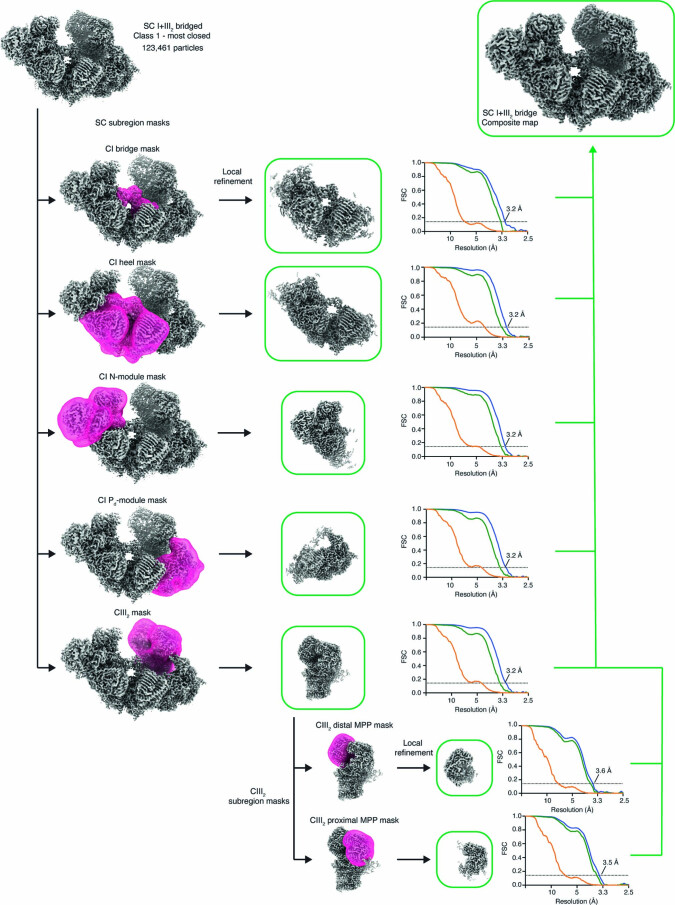
Focused refinements for SC I + III_2_ bridged class 1. Individual masked refinements using the masks shown in magenta were performed to improve the map quality. Fourier shell correlation (FSC) curves for the individual focused refinements are shown for no (orange), loose (green) and tight (blue) masking. The resolution at which the tight mask FSC crosses the 0.143 gold-standard limit (dashed line) is indicated. The focused maps highlighted with green boxes were combined into the final composite map of the bridged SC I + III_2_ (top left).

Our cryoEM image processing resulted in two initial reconstructions of SC I + III_2_ containing the full complement of subunits (‘bridged’ SC I + III_2_) at 3.3 Å and 3.6 Å resolution (class 1 and class 2, respectively) (Extended Data Fig. 2 and Extended Data Tables 1 and 2). Three-dimensional variability analysis (3DVA) of all bridged SC I + III_2_ particles demonstrated that most variability in the bridged particles stemmed from the flexible interface between CI and CIII_2_ (Supplementary Movies 1 and 2), resulting in an overall lower-quality map for CIII_2_ in both classes (Extended Data Fig. 2). To overcome the inherent flexibility of the particles, we performed focused refinements on the bridged SC I + III_2_ class 1 particles using masks around CI’s bridge domain, CI’s ‘heel’, CI’s N-module, CI’s distal pump (P_d_) module and CIII_2_ (Extended Data Fig. 3 and Extended Data Table 1). Further improvement to the CIII_2_ map quality was achieved by subsequent focused refinement around the MPP domains (Extended Data Fig. 3 and Extended Data Table 1). These focused refined maps were combined into a composite map with nominal resolution of 3.2–3.6 Å (Fig. 1b, Extended Data Fig. 3 and Extended Data Table 1). In addition to the bridged classes, less populated classes were identified for SC I + III_2_ missing the ferredoxin bridge and other subunits (‘bridge-less’ SC I + III_2_) and for CI alone (Extended Data Fig. 2 and Extended Data Table 2). The bridge-less particles could be classified into four classes, further discussed below. The reconstruction of isolated CI lacked clear density for the bridge, NDUA11 and TMHs in Nad4-Nad6, and showed poor density for the P_d_ module, consistent with previously published structures of plant CI (refs. 10, 11). This suggests that, in plants, CIII_2_ may be needed to stabilize intact CI after extraction from the membrane. How the assembly and integrity of each complex may affect that of the other has been studied in mammals^16^, but remains to be fully investigated in plants.

**Extended Data Fig. 4:**
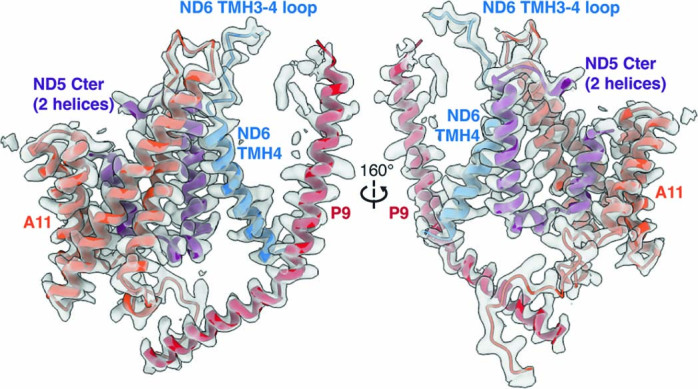
Density for the CI subunits or subunit fragments that were improved in this atomic model compared to previous structures of plant CI. Subunits shown in coloured cartoons over semi-transparent density in grey.

We built the atomic model for *V. radiata* SC I + III_2_ (Fig. 1b–e) on the basis of previously published structures of plant CI and CIII_2_ (refs. 9, 11, 15). The improved density of the map relative to previous reconstructions for *V. radiata* CI fragment and CIII_2_ allowed us to determine several additional C-to-U RNA editing sites in Nad1, Nad2, Nad3, Nad4, Nad4L, Nad5, Nad6, NDUS2 and NDUS3 (refs. 9, 15, 17) (Extended Data Table 3). The SC I + III_2_ map also contained density for subunits or regions of subunits at the interface between CI and CIII_2_ that were previously missing in other CI structures. These were the accessory subunit NDUA11 (B14.7), the C-terminus of core subunit Nad5, the fourth transmembrane helix (TMH4) of core subunit Nad6 and the loop between Nad6’s TMH3 and TMH4 (Fig. 1c and Extended Data Fig. 4). This confirmed that NDUA11 is a bona fide CI subunit in plants and established that Nad5’s C-terminus contains two TMHs, as in yeast^18^. The positions of Nad6’s TMH4 and TMH3-4 loop have functional implications discussed below. Additionally, the map contained an extended L-shaped density for a subunit in the vicinity of NDUA11 that remained unidentified in previous plant CI reconstructions^9–11^. This L-shaped density extends into the interface between CI and CIII_2_, providing inter-complex contacts not seen in other species (Fig. 1c). Using the density to obtain a preliminary amino acid sequence followed by a BLAST search, we were able to assign the subunit as the product of *V. radiata* gene *LOC106767179* (homologue of *A. thaliana* *At1g67785*). This corresponds to a CI-subunit candidate as determined by mass spectrometry studies^19–21^ that was initially erroneously assigned as NDUB5 (ref. 22) and later renamed P4 (ref. 23). However, given that the alga *Chlamydomonas reinhardtii* and *Polytomella* sp. contain established complex I subunits P4–P8 (refs. 11, 24), to avoid confusion we propose to call this subunit NDUP9. This subunit appears to be plant specific, as standard BLAST searches returned only plant species. Overall, this SC I + III_2_ map provides the full complement of plant CI subunits (14 core and 34 accessory; Extended Data Table 3).

**Extended Data Fig. 5:**
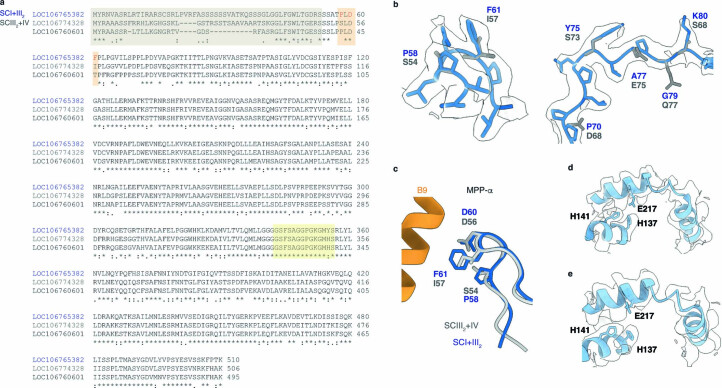
MPP-α isoform in *V. radiata* SC I + III_2_. (**a**) Sequence alignment of MPP-α isoforms annotated in *V. radiata* proteome. Isoform modelled into SC I + III_2_ (LOC106765382) in blue, Isoform modelled into SC III_2_ + IV (corresponding to *LOC106774328*) in gray. Transparent brown box, signal sequence. Transparent orange box, interface residues with CI’s NDUB9. Residues that differ marked in red font. Transparent yellow box, gly-rich loop involved in MPP substrate recognition. (**b**) Key regions of map-to-model fit of the MPP-α subunits modelled for SC I + III_2_ and SC III_2_ + IV, fit into the SC I + III_2_ map. LOC106765382 (SC I + III_2_) in blue, LOC106774328 (SC III_2_ + IV) in gray. (**c**) MPP-α interface with CI’s NDUB9. Protein corresponding to *LOC106765382* (SC I + III_2_) in blue and *LOC106774328* (SC III_2_ + IV) in gray. (**d-e**) MPP-β catalytic triad in protomer proximal to CI (d) and distal to CI (e). Note the lack of density for a putative Zn^2+^.

The SC I + III_2_ map also revealed differences in CIII_2_’s MPP domain compared with the previously available structures from *V. radiata*’s supercomplex III_2_ + IV (SC III_2_ + IV) and CIII_2_ alone^15^. Plants contain multiple isoforms of MPP-α (Extended Data Fig. 5a). We previously determined that in SC III_2_ + IV both MPP-α subunits correspond to gene *LOC106774328* (ref. 15). In contrast, in SC I + III_2_, the MPP-α subunit in the CIII_2_ protomer closest to CI was a different isoform (corresponding to gene *LOC106765382*), as revealed by the better fit of this protein to the density of SC I + III_2_ (Extended Data Fig. 5b). For MPP-α in the other protomer of SC I + III_2_, the density is ambiguous, with some positions more closely fitting the *LOC106765382* sequence and some positions more closely fitting the *LOC106774328* sequence. This suggests that the density might represent the average of a mixed population, or that databases may be mis-annotated. The main differences between isoforms are in the N-terminus, including a short region that interacts with CI (see below). The functional relevance of these differences in MPP-α remains to be explored. We also observed differences in MPP-β. While the MPP-β isoforms were the same as those in SC III_2_ + IV, the helix containing the catalytic glutamate (Glu217) was partially disordered in both protomers in SC I + III_2_ (Extended Data Fig. 5d,e). This leads to an inability to coordinate the catalytic Zn^2+^ (Extended Data Fig. 5d,e), which would render the MPP domain non-functional. Whether this loss is a biologically relevant consequence of SC I + III_2_ formation remains to be determined. Alternatively, the loss of Zn^2+^ could be a consequence of our purification procedure, which was slightly different from that used to obtain the SC III_2_ + IV sample in which Zn^2+^ was seen in the MPP active sites^15^. It will be interesting to compare these findings on *V. radiata* SC I + III_2_ with those from other plant species.

## *V. radiata* SC I + III_2_ interfaces differ from those of other organisms

**Fig. 2:**
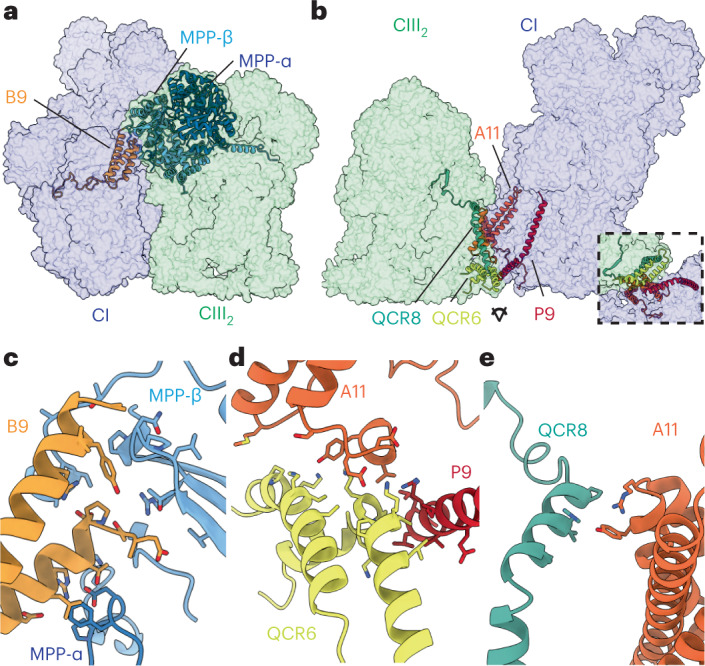
SC I + III_2_ interfaces in *V. radiata*. **a**,**b**, *V. radiata* SC I + III_2_ matrix (**a**), membrane and intermembrane space (IMS) interfaces (**b**). Interacting subunits shown as coloured cartoons over semi-transparent complex I (CI) surface (blue) or complex III_2_ surface (green). Inset in **b** shows interface viewed from the IMS. **c**–**e**, *V. radiata* interaction details for matrix (**c**), IMS (**d**) and membrane (**e**) interfaces. Subunits shown as coloured cartoons with key residues as sticks coloured by atom. Some structural elements are hidden for clarity.

**Extended Data Fig. 6:**
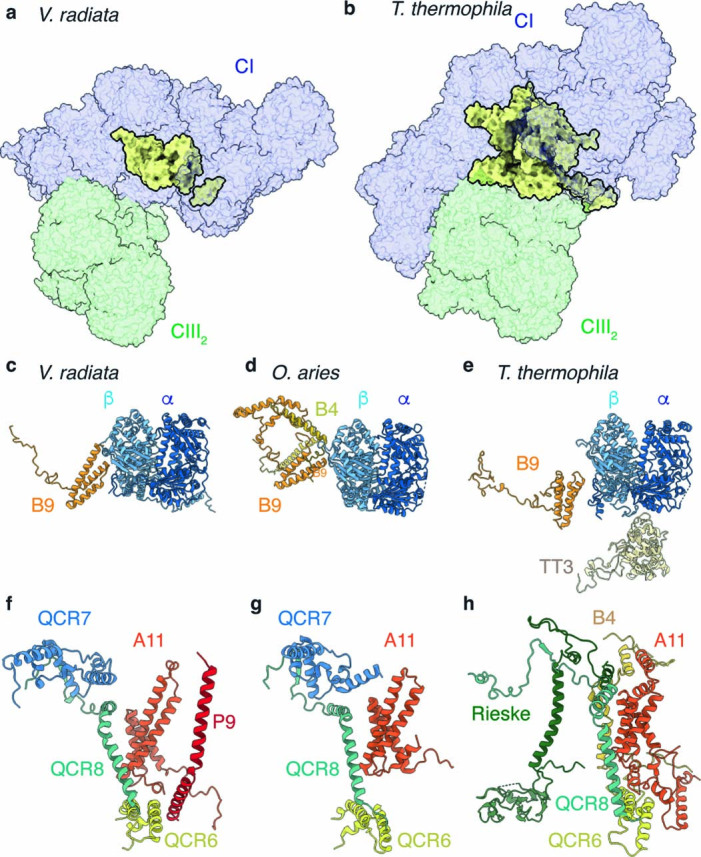
Inter-species comparison of SC I + III_2_. (**a-b**) Comparison of ferredoxin bridge (yellow surface) in *V. radiata* (a) and *T. thermophila* (b) (PDB 7TGH^13^,). Supercomplexes aligned by CIII_2_. CI and CIII_2_ coloured as in (a). (**c-h**) Inter-species comparison of SC I + III_2_ interfaces in *V. radiata* (c,f), *O. aries* (d,g PDB 6QC5^12^,) and *T. thermophila* (e,h). The main interacting subunits are shown in cartoon. (**c-e**) Matrix interfaces involving MPP-α (α) and MPP-β (β), aligned by MPP-β. (**f-h**) Membrane and inter-membrane space interfaces, aligned by NDUA11. Note that *V. radiata* QCR7 is shown for comparison but does not participate in the interface.

The mung bean SC I + III_2_ map showed three interfaces between CI and CIII_2_: (1) a matrix site formed between NDUB9 (CI), MPP-β (CIII_2_) and MPP-α (CIII_2_), (2) an IMS site formed between NDUP9 (CI), NDUA11 (CI) and QCR6 (CIII_2_) and (3) a membrane site between NDUA11 (CI) and QCR8 (CIII_2_) (Fig. 2a–e). Contrary to *T. thermophila*’s SC I + III_2_, where the CI bridge domain provides an extensive SC interface^13^, *V. radiata*’s CI bridge domain did not directly participate in the formation of this supercomplex (Extended Data Fig. 6a,b). The MPP domain of CIII_2_ provided the largest interface, mainly through hydrophobic and electrostatic interactions between MPP-β and NDUB9 (Fig. 2c). MPP-α also interacts with NDUB9 via three amino acids in MPP-α’s N-terminal extension, two of which differ between the MPP-α isoforms. When comparing MPP-α’s N-terminal extension from the SC I + III_2_ structure (*LOC106765382*) with that in SC III_2_ + IV (*LOC106774328*), we observed that the loops occupy roughly the same position (Extended Data Fig. 5c). Moreover, the aspartate residue that is positioned to form a salt bridge with B9-Arg64 is conserved in both MPP-α isoforms. Given the structural similarity, the conservation of the aspartate and the minimal contribution of MPP-α to this interface, it is unlikely that this loop of MPP-α would suffice to differentially regulate the formation of SC I + III_2_ versus SC III_2_ + IV. Nevertheless, the hypothesis that CIII_2_ with different MPP-α isoforms is selectively incorporated into different CIII_2_ supercomplexes remains to be experimentally tested. In the IMS, NDUP9 and NDUA11 contacted QCR6 (Fig. 2d). While NDUP9 and QCR6 showed mostly hydrophobic interactions, those between QCR6 and NDUA11 were more electrostatic in nature. Lastly, the membrane site was a small interface of a couple of residues on NDUA11 and QCR8 (Fig. 2e). As in the *O. aries* and *T. thermophila* SC I + III_2_ interactions, these three sites in *V. radiata* were linked from the IMS to the matrix via QCR8’s participation in MPP-β’s anchoring β-sheet.

Although the mung bean SC I + III_2_ interfaces were reminiscent of those seen in *O. aries* and *T. thermophila* (for example, involving NDUB9, NDUA11 and QCR8 in all three organisms), the specific protein and amino acid interactions were different (Extended Data Fig. 6c–h). SC interactions through NDUP9 appear to be plant specific. Furthermore, superpositions of *V. radiata*, *T. thermophila* and *O. aries* SC I + III_2_ showed that the angle of approach between CI and CIII_2_ differed between organisms, as seen in the subtomogram averages^7^. A comparison of the CI:CIII_2_ interactions and orientation with other plant species will determine whether the interfaces observed here are common across plant families.

## *V. radiata* CI shows a non-canonical A/D response

In several organisms, CI has been seen to adopt multiple conformations based on the hinging between its membrane and peripheral arms^18,25–28^. Analogous conformations have been seen for CI in the mammalian SC I + III_2_, where it can adopt a distinct ‘closed’ state and an ensemble of ‘open’ states^12^. These large-scale changes are accompanied by conformational changes in loops in the vicinity of the quinone-binding site (Nad1 TMH5-6, NDUFS2 β1-2 loop, and NDUFS7 α1-2 loop and α2-β1) and at the interface between CI’s arms (Nad3 TMH2-3 and Nad6 TMH3-4) (refs. 18,25–28). Furthermore, in some organisms CI can undergo an A/D transition^29–31^. This is an off-pathway transition that places CI in a catalytically incompetent state when in the absence of substrate, protecting against ischaemic reperfusion injury in mammals^30–34^. Given that the A/D transition also occurs through conformational changes, it has remained controversial whether CI’s open and closed conformations correspond to intermediate states in CI’s catalytic cycle or to its active and deactive states^28,35–37^. In plants, the presence of the A/D transition has not yet been investigated. Therefore, we examined the large-scale and loop conformations of *V. radiata*’s CI in the SC I + III_2_ structure, as well as CI’s ability to undergo the A/D transition in mitochondrial membranes.

### Large-scale changes of CI within SC I + III_2_

**Fig. 3:**
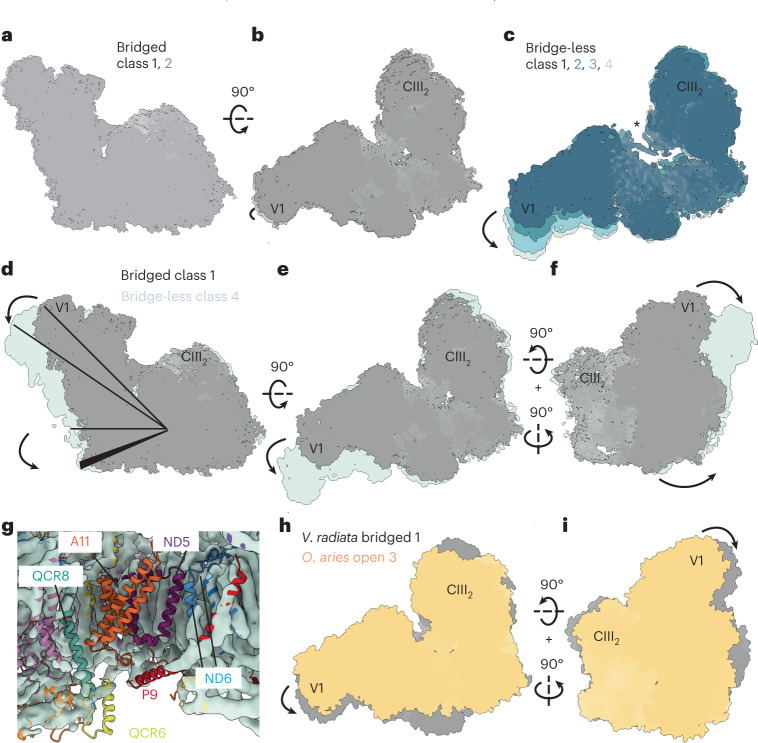
Large-scale conformational changes of complex I within *V. radiata* SC I + III_2_. **a**–**c**, Bridged (**a**,**b**) and bridge-less classes of SC I + III_2_ viewed from the side (**a**) or the matrix (**b**,**c**). Bridge class 1 in grey, class 2 in lighter grey. Bridge-less class 1 through class 4 in progressively lighter shades of blue. The asterisk indicates the presence of NDUA11 in bridge-less class 1. **d**–**f**, Differences between bridged and bridge-less classes of SC I + III_2_ viewed from the side (**d**), matrix (**e**) or the back of CI from the plane of the membrane (**f**). Bridged SC I + III_2_ class 1 shown in grey aligned with bridge-less class 4 in light blue. Rotations are indicated with respect to **d**. The opening of the peripheral arm and the straightening of the membrane arm are represented with lines and arrows. **g**, CI and CIII_2_ subunits and fragments that are lost in the bridge-less class 4 shown in coloured cartoons over light-blue surface of class 4 map. **h**,**i**, Comparison of *V. radiata* bridge class 1 (grey) and the most open SC I + III_2_ class from *O. aries* (PDB: 6QC4) (ref. 12) (light orange) viewed from the matrix (**h**) or the back (**i**). The positions of complex III_2_ (CIII_2_) and CI’s subunit NDUFV1 (V1) are shown for orientation.

Our cryoEM image processing identified six distinct three-dimensional classes: two major classes of the bridged SC I + III_2_ described above (~72% of supercomplex particles) and four minor bridge-less classes (~28% of supercomplex particles) varying in conformation and composition (Fig. 3 and Extended Data Fig. 2). The bridged classes showed only a small difference in their overall CI conformations (Fig. 3a,b and Extended Data Movies 1 and 2). In contrast, the bridge-less classes showed a progressive increase in the angle between CI’s peripheral and membrane arms and a decrease in the curvature of CI’s membrane arm, both between themselves as well as compared with the bridged classes (Fig. 3c–f). The bridge-less classes not only lost the bridge-domain subunits (NDUFX, NDUA6 and NDUAB1-α) but also progressively lost density for subunits or subunit segments at the interface between CI and CIII_2_ (NDUA11 subunit, NDUP9’s C terminus, Nad5’s two C-terminal helices, Nad6’s TMH4, Nad6’s TMH3-4 loop, QCR6’s C-terminus and QCR8’s transmembrane helix) (Fig. 3g). Given the importance of some of these subunits for catalysis, it is unlikely that the bridge-less SC I + III_2_ classes are functional, or that the loss of the bridge is a regulatory process as previously suggested for *A. thaliana* CI (ref. 11). In our view, bridge-less classes are more likely the product of progressive degradation during sample purification and/or cryoEM grid preparation.

We also compared the angle between CI’s arms in *O. aries* and *V. radiata* SC I + III_2_. Although *V. radiata*’s bridged SC I + III_2_ was more closed (smaller angle between the arms) than bridge-less SC I + III_2_, it was still more open than the most open *O. aries* SC I + III_2_ (Fig. 3h,i). Therefore, it is unclear from the angle alone whether *V. radiata*’s bridged SC I + III_2_ should be considered to have CI in an ‘open’ or a ‘closed’ state.

### Conformational changes to CI loops

To obtain more clarity on the state of CI in *V. radiata*’s SC I + III_2_, we compared the features of its quinone and interface loops to those of CI in previously observed states, for example, open and closed classes in *O. aries*^26^, the deactive state in *Mus musculus*^25,27^ and *Yarrowia lipolytica* CI in turnover and deactive states^18^.

**Fig. 4:**
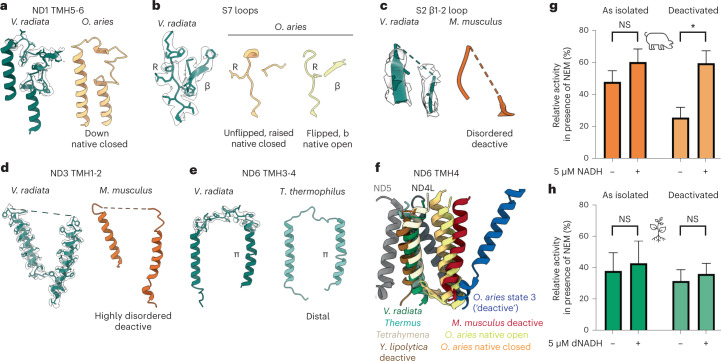
Conformational state of complex I’s catalytic loops. **a**–**e**, Species comparison of CI’s loops associated with catalysis, showing the *V. radiata* model and associated density (green cartoon, transparent map) (left) and the model for the structure it most resembles, coloured by structure (right). **a**, Nad1 TMH5-6 loop; *O. aries* native closed (light orange), PDB: 6ZKO^26^. **b**, NDUFS7 α1-2 loop (left) and α2-β1 loop (right). Key arginine residue (R) and β-strand are marked; *O. aries* native closed class with ‘unflipped’ arginine in β1-2 loop, β-strand for the α2-β1 loop (light orange, left), PDB: 6ZKO^26^ and *O. aries* native open with flipped arginine and β-strand (yellow, right), PDB: 6ZKP^26^. **c**, NDUFS2 β1-2 loop; *M. musculus* deactive (dark orange), PDB: 6G72 (ref. 25). **d**, Nad3 TMH1-2 loop; *M. musculus* deactive (dark orange), PDB: 6G72 (ref. 25). **e**, Nad6 TMH3-4 loop; *Thermus thermophilus* native (light teal), PDB: 4HEA^64^. **f**, Position of Nad6 TMH4 across organisms and conditions. Structures aligned by Nad6. The *V. radiata* Nad5 TMH16 and Nad4L TMH1 shown for orientation (grey cartoon). Structure: *V. radiata* (green, this study), *T. thermophilus* native (light teal, PDB: 4HEA^64^), *Tetrahymena thermophila* native (cream, PDB: 7TGH^13^), *Yarrowia lipolytica* deactive (brown, PDB: 7O71 (ref. 18)), *O. aries* native closed (light orange, PDB: 6ZKO^26^), *O. aries* native open (yellow, PDB: 6ZKP^26^), *M. musculus* deactive (red, PDB: 6G72 (ref. 25)), *O. aries* ‘deactive’ (blue, PDB: 6KZS^26^). **g**,**h**, A/D transition in *S. scrofa* (**g**) and *V. radiata* (**h**) mitochondrial membranes. Membranes were treated with 2 mM NEM as isolated (orange, green) or after thermal deactivation (light orange, light green), in the presence or absence of pre-activation with 5 μM NADH (*S. scrofa*) or 5 μM dNADH (*V. radiata*). Values are the percentage of average activities (NEM/no NEM) determined from four to ten independent measurements on single samples of isolated *S. scrofa* or *V. radiata* mitochondrial membranes shown in Extended Data Fig. 7. Error bars equal the coefficient of variation for the ratio calculated as the sum of the coefficient of variation of the individual rates multiplied by the value of the ratio. Statistical significance of the difference between the ratios was determined using a two tailed *z*-test. *P < 0.05 (*P* = 0.02 for deactivated *S. scrofa*). NS, not statistically significant (*P* > 0.05).

**Extended Data Fig. 7:**
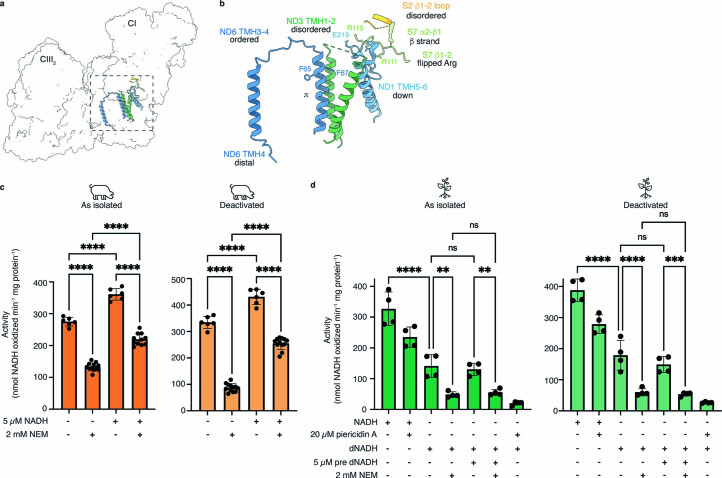
Further detail on *V. radiata* CI loops and active-to-deactive (A/D) transition. (**a-b**) Summary of CI loop conformations. (a) Key loops, helices and β-strands discussed in text shown in coloured cartoon over SC I + III_2_ transparent surface. The inset (dashed square) is shown in detail in (b). Configuration of each loop, as well as key residues and features are marked. (**c-d**) Activity rates for the A/D transition in porcine (*S. scrofa*, pig symbol) (c) and *V. radiata* (plant symbol) membranes (d). Activity in the presence or absence of 2 mM NEM, 20 μM piericidin A, pre-activation with 5 μM NADH or 5 μM dNADH, and thermal deactivation is shown for 4-12 repeats. Data are shown as individual values and mean ± standard deviation. Statistical analysis with one-way ANOVA with Šídák’s multiple comparisons test. **, *p* < 0.01; ****, *p* < 0.0001. Data is representative of two fully characterized (NADH and dNADH) *V. radiata* mitochondrial membrane isolations.

Broadly speaking, it has been seen in multiple organisms that loops become ordered in the closed or turnover states and disordered in the open and deactive states. Nevertheless, the *V. radiata* loops did not fully coincide with any single previously observed state (Fig. 4a–e and Extended Data Fig. 7a,b). In the quinone-binding region, *V. radiata*’s Nad1 TMH5-6 loop was ordered in what resembled the ‘down’ conformation of the closed class of ovine CI, also seen in the yeast turnover and deactive states (Fig. 4a). However, not all key glutamate residues (*V. radiata* Nad1-Glu207, Glu209 and Glu219) were within appropriate distance to form salt bridges with key arginine residues in S7 (*V. radiata* S7 Arg111 and Arg115) as seen in the ovine closed, yeast turnover and other structures^18,26,38^. *V. radiata*’s Nad1 TMH5-6 loop was not equivalent to the ordered loop in the recently observed ‘open-ready’ state of the *Escherichia coli* CI either^37^, as *V. radiata*’s Nad1-Glu211 pointed away from the S2 β1-2 loop. Furthermore, key residue S7 Arg111 was in an ‘unflipped’ position similar to the ovine closed and the yeast in turnover or deactive states, but with a β-strand for residues S7-Pro81-Leu86 typical of the ovine open and the yeast deactive states (Fig. 4b). The S2 β1-2 loop was disordered, in accordance with the ovine open class and the murine deactive state, but not the yeast deactive state (Fig. 4c). As for the loops at the interface between CI’s arms, the Nad3 TMH1-2 loop was disordered across its entire length, resembling the murine deactive state (Fig. 4d). *V. radiata*’s Nad6 TMH3-4 loop was ordered, but the Nad6 TMH3 showed the characteristic π bulge seen in the open and deactive mammalian structures as well as in the deactive and turnover yeast structures (Fig. 4e). Importantly, *V. radiata* Nad6’s TMH4 was in a ‘distal’ position at the interface between Nad4L’s TMH1 and Nad5’s TMH16 (Fig. 4f). This is ~14 Å away from the position of Nad6 TMH4 in the ovine open/closed classes, ~16 Å away from the murine deactive state and ~25 Å away from the tilted Nad6 TMH4 in the ovine ‘deactive’ state^26^ (which lacks density for NDUFA11 and ND5 and is thus more similar to what has been called ‘state 3’ CI, that is, in initial stages of degradation^28^). Rather, *V. radiata* Nad6 TMH4’s position most closely resembled that in *Thermus thermophilus* with or without substrates^39,40^ as well as that in *E. coli* in open, open-ready or closed structures^37^, with similarities to the *Tetrahymena thermophila* structure^13^ and the *Y. lipolytica* deactive state^18^ (Fig. 4f).

These findings indicate that the loop conformations in our *V. radiata* structure cannot be neatly correlated with previously described CI open or closed states, nor are they fully equivalent to the deactive state in mammals or yeast. Nevertheless, the presence of the π bulge in Nad6’s TMH3, which rotates hydrophobic residues into CI’s hydrophilic axis and interrupts the water wire thought to be required for proton pumping^26,35^, suggests that our structure contains CI in a resting state.

### A/D transition

To our knowledge, the existence of the A/D transition has not been previously investigated in plants. Therefore, we tested the ability of *V. radiata* CI to undergo the A/D transition using a standard assay with isolated mitochondrial membranes^30,31,41,42^. In this assay, CI is deactivated by incubating the membranes at 37 °C in the absence of substrate, leading to the disordering of the active site and potential large-scale opening of the structure^27^. Then, *N*-ethylmaleimide (NEM) is added to ‘trap’ CI in the deactive conformation by modifying a conserved cysteine in Nad3’s TMH1-2 loop (Cys44 in *V. radiata*), which is exposed in the deactive state but inaccessible in the active state. Hence, NEM modification prevents re-activation of CI, leading to a reduction in the observed NADH oxidation rate. The detrimental effects of NEM can be minimized by pre-activating the complex with a low concentration of NADH (substrate) before incubation with NEM.

We compared *V. radiata*’s response to the A/D assay with that of mammalian mitochondrial membranes isolated from *Sus scrofa* (pig), whose CI is known to undergo a ‘classic’ A/D transition^30^. As expected, exposure of porcine mitochondrial membrane to 2 mM NEM decreased CI rates, both for thermally deactivated and for ‘as isolated’ (not deactivated) membranes. The NEM effect was partially rescued by pre-activation with 5 μM NADH in both cases. Moreover, the inhibitory effect of NEM was higher in deactivated membranes than in non-deactivated membranes, as expected (Fig. 4g and Extended Data Fig. 7c).

We performed similar assays with *V. radiata* mitochondrial membranes. To preclude the effects of plants’ mitochondrial alternative NADH dehydrogenases, we used the NADH analogue deaminoNADH (dNADH), which can be used as a substrate by CI but not by the alternative dehydrogenases^43–46^ (Extended Data Fig. 7d). Although *V. radiata* membranes were also susceptible to NEM, their susceptibility did not show major changes upon thermal deactivation (Fig. 4h and Extended Data Fig. 7d). This is consistent with the incubation without substrates at an increased temperature not leading to increased exposure of Nad3’s THM1-2 loop. Additionally, *V. radiata*’s susceptibility to NEM did not change upon pre-activation with 5 μM dNADH (Fig. 4h and Extended Data Fig. 7d). That is, plant CI was just as susceptible to NEM whether the enzyme had recently turned over or not. This suggests that plant CI does not enter biochemically distinct states equivalent to the A and D states seen in mammalian CI, possibly owing to the presence of the bridge domain.

Overall, *V. radiata*’s CI in SC I + III_2_ exhibited non-canonical characteristics in terms of its large-scale structure, its catalytic loops and its behaviour in the standard A/D assay.

## Discussion

Here we present the cryoEM structure of active SC I + III_2_ purified from *V. radiata* mitochondria, together with a functional examination of the A/D transition in isolated mitochondrial membranes.

The SC I + III_2_ assembly protected several CI subunits at the interface with CIII_2_. Therefore, our reconstruction contained the full structure of CI, including accessory subunit NDUA11, additional TMHs of core subunits Nad5 and Nad6, as well as a newly assigned, probably plant-specific CI subunit NDUP9 (Fig. 1 and Extended Data Figs. 1–3). Our structure also contained a different isoform of MPP-α in one CIII_2_ protomer relative to that seen in SC III_2_ + IV (corresponding to genes *LOC106765382* and *LOC106774328*, respectively; Extended Data Fig. 5). This implies that CIII_2_ supercomplexes may be differentially assembled depending on the MPP-α isoform present. Furthermore, the lack of catalytic Zn^2+^ in both MPP-β subunits suggests that CIII_2_’s MPP function is not active when CIII_2_ is assembled in SC I + III_2_. To our knowledge, the potential functional differences of MPP-α or MPP-β isoforms have not been investigated. In potato, MPP-α1 is more highly expressed at the messenger RNA level across several mature plant tissues than MPP-α2, without substantial tissue-specific variation under standard conditions^47,48^. Additionally, in *A. thaliana*, MPP-α2 (At3g16480) is dually targeted to chloroplasts and mitochondria^48^. Standard sequence alignments between the *V. radiata* and *A. thaliana* MPP-α isoforms did not conclusively reveal the homology relationships between the isoforms. The hypotheses that MPP isoforms sort CIII_2_ into different supercomplexes, that CIII_2_’s MPP is active in SC III_2_ + IV but not in SC I + III_2_ and that supercomplexes are differentially assembled in certain tissues, stresses or developmental stages remain to be experimentally tested.

As expected from low-resolution tomographic comparisons^7^ and sequence alignments, the details of the SC I + III_2_ interface are plant specific (Fig. 2). The main interface in *V. radiata* is on the matrix side, between MPP-β and NDUFB9, which might restrict the flexibility and function of the MPP domain. A key feature of the SC I + III_2_ interface in plants is the participation of the new CI subunit, NDUP9. The functional analysis of NDUP9 mutants will illuminate the physiological roles of SC I + III_2_ and supercompex formation in general, as has begun to be investigated with NDUFX and NDUA11 mutants^49^.

Specific details notwithstanding, the broad arrangement of the complexes in SC I + III_2_ is conserved between plants, mammals^12^, alveolates^13^ and yeast^7^. This suggests that this supercomplex arrangement provides evolutionary advantages, potentially through the stabilization of subunits or through the balancing of electron flux to limit reactive oxygen species formation. For instance, by ensuring that CI and CIII_2_ are in vicinity to each other, the formation of SC I + III_2_ could preclude the generation of local differences in the quinone-to-quinol ratio, especially between mitochondrial cristae, which could otherwise promote the production of reactive oxygen species in regions where the pool is over reduced^50^.

Like in other organisms, our cryoEM processing yielded multiple classes for SC I + III_2_ (refs. 11, 12, 25, 26). However, rather than differing mainly in the angle of CI, they also differed in the compositional integrity of CI and increased flexibility of CIII_2_ (Fig. 3 and Extended Data Fig. 2). Four of the six classes displayed loss of the bridge domain (‘bridge-less’ SC I + III_2_). This was accompanied by loss or disordering of accessory and core subunits (NDUA11 lost; NDUP9, QCR6 and QCR8, Nad5 and Nad6 partially disordered) as well as by increases in the angle between CI’s arms and between CI and CIII_2_. The two ‘bridged’ classes contained good density for the bridge domain and differed only slightly in the angle between the CI arms. These findings imply that the bridge restrains the flexibility of CI in plants, limiting the angle and range of motion of the arms and helping maintain the enzyme’s compositional integrity. Furthermore, NDUA11, Nad5’s C-terminus and Nad6’s TMH4 are critical for catalysis, and their loss and increased flexibility have been noted in structures of mammalian CI in the initial stages of degradation (‘class/state 3’) (refs. 25, 28, 51, 52). Therefore, in our view, the loss of the bridge and associated subunits and the concomitant increase in CI’s angle in the bridge-less classes more likely reflect a progressive degradation of the sample during preparation than a regulatory mechanism for CI (ref. 11). Our view is in line with recent functional analyses of *A. thaliana* knockout lines of NDUFX (one of the bridge subunits)^49^, which lack SC I + III_2_. Despite the lack of the bridge and of SC I + III_2_, these mutants do not exhibit growth or developmental defects under standard conditions, which would be expected if the bridge domain regulated CI’s activity. Moreover, given that the NDUFX mutants partially accumulate CI at the CI* assembly-intermediate stage rather than in bridge-less supercomplexes, it is also unlikely that our bridge-less particles originated from assembly defects^49^. Instead, the CI bridge probably plays a structural role in keeping CI’s arms in a permissive state for SC I + III_2_ formation^49^. Further experiments on the role of the bridge in CI assembly and supercomplex formation are needed.

Although the bridged classes appeared ‘closed’ (smaller angle) relative to the bridge-less classes, they were more open than the most open mammalian SC I + III_2_ class (Fig. 3). This implies that the open and closed denominations and angles are relative, and supports the view that the opening and closing of the structure may simply reflect the organism-specific inherent flexibility of the complex^38^. The fact that the bridge domain in plants limits CI’s conformational flexibility and that plant CI cannot adopt more open states without losing the bridge suggests that changes in the angle between the arms are not part of CI’s conserved catalytic mechanism. Moreover, our structure shows that opening and closing of the angle is not necessary for the ordering or disordering of the catalytically relevant loops in plants. This is in line with the observations from *T. thermophila*’s CI (ref. 13), which also contains a bridge domain, as well as from *Chaetomium thermophilum*’s CI (ref. 38), which does not contain a ferredoxin bridge but does have additional bridging interactions via an extension on NDUA5. It also agrees with recent structures of *E. coli* CI under different conditions, which show differences in the rotation of the PA but not in the angle between the arms^37^. Nonetheless, it remains to be examined whether the angle of *V. radiata*’s (as well as *T. thermophila*’s and *C. thermophilum*’s) CI changes under turnover conditions. Regardless, it is increasingly clear that the distinctions between CI states should focus on the conformation of the relevant loops and helices rather than on the large-scale angles between the arms, as recently suggested by others^37,38^.

Our examination of CI’s catalytic loops revealed that *V. radiata*’s CI (isolated in the absence of substrates) contained a mixture of features previously seen in open/closed and turnover/native/deactive states without neatly aligning with any single state (Fig. 4 and Extended Data Fig. 7). The quinone-binding site appeared in a semi-ordered state with the Nad1 TMH1-2 loop in a down position. However, only one glutamate (Glu219) in Nad1 TMH1-2 was within salt-bridging distance to key arginine residues in the S7 β1-2 loop (Arg115), even though Arg111 faced towards the Nad1 TMH1-2. The rest of the quinone site loops (S7 α2-β1 loop and S2 α1-2 loop) were reminiscent of CI in deactive and open states^25,26^. Among the ‘interface’ loops, the highly disordered Nad1 TMH1-2 loop was reminiscent of the deactive murine state and Nad6 contained a π bulge typical of deactive but also some substrate-bound structures^18,25,26^. The position of Nad6’s TMH4 was ‘distal’, that is, tucked in between Nad5 and Nad4L, reminiscent of non-mammalian structures in multiple conditions, and very different from the position in mammalian deactive structures^13,18,25,26,37,39^. Together, this suggests that the resting conformation of the enzyme is in an ‘intermediate’ state relative to the previously observed states in other organisms.

The non-canonical loop configuration is in line with our results from the A/D assays, where *V. radiata* membranes were susceptible to NEM inhibition, but their susceptibility (that is, the accessibility of the Nad3 TMH1-2 loop) did not change upon either incubation without substrate at 37 °C (‘deactivation’) or pre-activation with substrate (Fig. 4 and Extended Data Fig. 7). This implies that plant CI’s loops may sample a range of conformations along the catalytic continuum both in the absence and presence of substrate. Thus, plants may have a lower energetic barrier between the states along CI’s catalytic pathway, and a lack of open/closed and active/deactive states, at least as currently biochemically defined. Although small thermodynamic barriers between the A and D states have previously been shown for yeast^30^ and proposed for a mouse strain that harbours a P25L mutation in Nad6 TMH2 (ref. 41), these cases are not equivalent to our observations. In yeast and the P25L mouse, CI in the absence of substrate rapidly converts to a stable D state, with an almost complete susceptibility to NEM and protection offered by pre-activation, although not to the same level as wild type. In contrast, *V. radiata* CI in isolated membranes was ~40% in a non-susceptible ‘A-like’ state, with no protection by pre-activation. Therefore, plants add to the repertoire of observations that must be considered in open/closed, active/deactive discussions.

Overall, we found that *V. radiata* CI within SC I + III_2_ showed an intermediate loop configuration, a non-canonical A/D transition and very limited differences in the angle between CI’s arms in the presence of the bridge domain. Our current definitions and assays for CI structure and function may need updating in light of studies of organisms beyond traditional heterotrophic model systems. It will be interesting to continue examining the A/D transition and reverse electron transfer in plants using mitochondrial membranes, submitochondrial particles and reconstituted proteoliposomes^36^ from *V. radiata* and other plant species. It will also be important to study the structure of plant CI in supercomplexes under cycling conditions, to examine whether an opening and closing of CI’s arms is present. The further study of CI and its supercomplexes in plants and in diverse organisms across the tree of life will allow for a more nuanced understanding of enzyme’s mechanism, including both universal and clade-specific features.

### Concluding statement

This work is published together with a study on the cryo-EM structure of supercomplex I + III_2_ from *Arabidopsis thaliana* at 2 Å resolution^53^. No experimental data or manuscript versions were exchanged between the groups before the papers were accepted, such that the independent studies would better complement and validate one another.

## Methods

### Mitochondrial purification

*V. radiata* seeds were incubated in 1% (v/v) bleach for 20 min and rinsed until water achieved a neutral pH. Seeds were then imbibed with 6 mM CaCl_2_ solution for approximately 24 h in the dark. Seeds were sown on plastic trays layered between damp cheesecloth to a final density of 0.1 g cm^−2^ and sprouted in the dark at 20 °C for 5 days. Seeds were given 2 l of water on day 1 of sowing and an additional 0.5 l of water on day 2. On day 5, etiolated sprouts were harvested by separating hypocotyls from roots and cotyledons by hand. Hypocotyls were further processed for mitochondrial purification as previously described^9,15^. In short, hypocotyls were homogenized in Waring blender with homogenization buffer (0.4 M sucrose, 1 mM EDTA, 25 mM MOPS-KOH, 10 mM tricine, 1% w/v PVP-40, 8 mM cysteine and 0.1% w-v BSA, pH 7.8), filtered through several layers of Miracloth and centrifuged for 10 min at 1,000 *g* at 4 °C. The resulting supernatant was centrifuged again for 30 min at 12,000 *g* at 4 °C. The pellets were resuspended in wash buffer (0.4 M sucrose, 1 mM EDTA, 25 mM MOPS-KOH and 0.1% w/v BSA, pH 7.2) and centrifuged at 1,000 *g* for 5 min at 4 °C. Supernatant was centrifuged for 45 min at 12,000 *g* at 4 °C. The pellets were resuspended in wash buffer and loaded onto sucrose step gradients (35%, 55% and 75% w/v) and centrifuged for 60 min at 72,000 *g* at 4 °C. Sucrose gradients were fractionated with BioComp Piston Gradient Fractionator connected to Gilson F203B fraction collector following absorbance at 280 nm. Relevant mitochondrial fractions were pooled and diluted 1:5 v/v in dilution buffer (10 mM MOPS-KOH and 1 mM EDTA, pH 7.2). The sample was centrifuged for 20 min at 16,000 *g* and 4 °C. The pellet was resuspended in final resuspension buffer (20 mM HEPES, 50 mM NaCl, 1 mM EDTA and 10% v/v glycerol, pH 7.5) and centrifuged for 20 min at 16,000 *g* at 4 °C. The supernatant was removed, and the pellet (purified mitochondria) was aliquoted, frozen and stored at −80 °C.

### Mitochondrial membrane wash

All steps were carried out at 4 °C with pre-chilled materials. Frozen *V. radiata* mitochondrial pellets were thawed, resuspended in double-distilled water at 5 ml g^−1^ of pellet and homogenized with a Dounce glass homogenizer. Potassium chloride was added to the homogenate to a final concentration of 0.15 M and homogenized again. The homogenate was centrifuged at 45,000 *g* for 90 min. Pellets were resuspended and homogenized again in Buffer M (20 mM Tris, 50 mM NaCl, 1 mM EDTA, 10% v/v glycerol, 10 U ml^−1^ DNase I, 2 mM dithiothreitol and 0.002% phenylmethylsulfonyl fluoride (PMSF, pH 7.4). The homogenate was then centrifuged at 45,000 *g* for 90 min at 4 °C. Pellets were resuspended in 3 ml Buffer M per gram of starting material and homogenized again. The protein concentration of the homogenate was determined with a Pierce BCA assay kit and was diluted to a final concentration of 20 mg ml^−1^ in 30% (v/v) glycerol for storage at −80 °C.

### SC I + III_2_ purification

*V. radiata* washed mitochondrial membranes were thawed on ice. Membrane complexes were extracted by tumbling for 60 min at 4 °C with digitonin at a 4:1 (w/w) ratio and 1% (w/v) concentration in Buffer MX (30 mM HEPES, 150 mM potassium acetate, 10% v/v glycerol, 1 mM EDTA and 0.002% PMSF). The extract was then centrifuged at 25,500 *g* for 30 min at 4 °C and amphipathic polymer A8-35 was added to the supernatant stepwise to a final concentration of 0.5% (w/v) while tumbling for 40 min. Sample was then transferred to dialysis membranes with 12,000–14,000 Da cut-off and dialysed in dialysis buffer (30 mM HEPES, 150 mM potassium acetate, 1 mM EDTA, 5% glycerol (v/v) and 200 μM γ-cyclodextrin, at pH 7.7) for 3 h at 4 °C. The dialysis membrane was then transferred to second dialysis buffer (30 mM HEPES, 150 mM potassium acetate, 1 mM EDTA, 5% glycerol (v/v) and 0.04% (w/v) BioRad Bio-beads SM-2, at pH 7.7) and dialysed overnight (~16 h) at 4 °C. The sample was recovered from the dialysis membrane and concentrated with centrifugal protein concentrators with 100,000 Da molecular weight cut-off. The concentrate was loaded onto 20–45% (w/v) linear sucrose gradients (in 15 mM HEPES and 20 mM KCl, pH 7.8) produced using the factory settings of a BioComp Instruments gradient maker, and centrifuged for 23 h at 243,500 *g* at 4 °C. The gradients were then fractionated using a BioComp Piston Gradient Fractionator connected to Gilson F203B fraction collector following absorbance at 280 nm. Throughout the purification, the NADH-dehydrogenase activity of SC I + III_2_ was measured spectroscopically with a ferricyanide (FeCy) activity assay adapted from ref. 54 as previously described^9,15^. See details in next section.

For cryoEM grid preparation, relevant fractions of the sucrose gradient were pooled, buffer-exchanged to remove the sucrose and concentrated to final protein concentration of ~1.5 mg ml^−1^. To remove the sucrose, the pooled fractions were diluted into 30 mM HEPES, 150 mM potassium acetate, 1 mM EDTA and 0.002% PMSF, pH 7.7 and concentrated using centrifugal protein concentrators of molecular weight cut-off 100,000 Da. The dilution and concentration steps were repeated several rounds until the estimated final concentration of sucrose was <1% (w/v).

For full biochemical purification of SC I + III_2_, the sample was concentrated to a final volume of 200 µl and subjected to SEC. The sample was injected onto a Superose 6 10-300 column using a BioRad NGC system and BioFrac Fraction Collector. Absorbance at 280 nm and 420 nm was monitored for collection of relevant fractions. Selected SEC fractions were pooled and concentrated using centrifugal concentrators with 30,000 Da cut-off. Protein concentration was measured with a Pierce BCA assay kit and diluted to ~0.185 mg ml^−1^ in Buffer MX (see above) and 30% (v/v) glycerol. The sample was aliquoted and stored in liquid nitrogen.

### NADH-dehydrogenase in-gel activity assay with blue-native polyacrylamide gel electrophoresis (BN-PAGE)

Sample aliquots were mixed with 5–8 µl of loading dye (5% (w/v) Brilliant Blue G, 0.5 M amino cupric acid and 50% (v/v) glycerol), loaded onto hand-cast 3–12% Tris–glycine PAGE gels and run at 4 °C. The anode buffer was 25 mM Tris and 192 mM glycine at pH 8.3. The dark-blue cathode buffer was 25 mM Tris, 192 mM glycine and 0.02% Coomassie-blue G-250 (w/v); the light-blue cathode buffer was identical to dark-blue buffer except that it contained 0.002% Coomassie-blue G-250 (w/v). Gels were run at constant voltage in dark-blue buffer for 30 min at 150 V, then switched to light-blue buffer and run for an additional 2 h at 200 V.

The in-gel NADH-dehydrogenase activity assay was performed on the basis of ref. 55. The gels were incubated in 10 ml of reaction buffer (1.5 mg ml^−1^ nitrotetrazoleum blue in 10 mM Tris–HCl pH 7.4 and 150 µM NADH), rocked at room temperature for ~10 min while purple bands (indicating NADH-dehydrogenase activity) developed. Once bands were sufficiently developed, the reaction was quenched using a solution of 50% (v/v) methanol and 10% (v/v) acetic acid. After imaging, the gel was stained with Coomassie stain (0.1% w/v Coomassie Brilliant Blue R250, 10% v/v glacial acetic acid and 50% v/v methanol).

### Spectroscopic activity assays

Assays were performed using 96-well plates in a Molecular Devices Spectramax M2 spectrophotometer. The following specialized reagents and manufacturers were used as needed: NADH (MilliporeSigma), dNADH (nicotinamide hypoxanthine dinucleotide, MilliporeSigma) cyt *c* purified from equine heart (MilliporeSigma), FeCy (MilliporeSigma) decylubiquinone (DQ; Santa Cruz Biotechnology), antimycin A (MilliporeSigma), piericidin A (Cayman Chemicals), superoxide dismutase (MilliporeSigma) and KCN (Honeywell). NADH oxidation was measured at 340 nm; cyt *c* reduction was measured at 550 nm. The path length of our reaction in the 96-well plates, and the extinction coefficients of NADH and cyt *c* used in activity calculations were experimentally determined (see below). An extinction coefficient of 5.4 mM^−1^ cm^−1^ was used for NADH and dNADH; an extinction coefficient of 6.5 mM^−1^ cm^−1^ was used for reduced–oxidized cytochrome *c*. The path length of our assay was 0.531 cm. Measurements of initial rates were done in replicates (detailed below), averaged and background corrected. Figures show averages and standard error from the mean.

#### Experimental determination of cytochrome *c* and NADH extinction coefficient

To experimentally determine the extinction coefficient of cytochrome *c*, we performed standard curves for oxidized and reduced equine cytochrome *c*. Lyophilized cyt *c* was diluted in 20 mM HEPES, pH 7.4, 50 mM NaCl and 10% glycerol (v/v) to a stock concentration of 25 mM. Working concentrations were made by diluting the stock into 20 mM HEPES, pH 7.4, 50 mM NaCl and 10% glycerol (v/v) buffer over a range of 25–125 μM. To oxidize or reduce cytochrome *c*, 400 μM potassium FeCy or 2 mM sodium dithionite were added to the working concentrations of cytochrome *c*. To ensure the oxidation state of cytochrome *c* at each working concentration, we obtained spectral scans for 350–600 nm every 2 nm, inspected the traces and calculated the *A*_550 nm_/*A*_565 nm_ ratio. An *A*_550 nm_/*A*_565 nm_ ratio >9.0 was considered fully reduced. With these fully reduced and oxidized cytochrome *c* samples, we measured absorbance at 550 nm to create standard curves for reduced and oxidized cytochrome *c*. Measurements of standard curve were done in three replicates with five cytochrome *c* concentrations, averaged and background corrected. The standard deviation was used to calculate the error. By measuring the slope, we determined an extinction coefficient of 12.7 mM^−1^ for reduced cytochrome *c* and 6.2 mM^−1^ for oxidized cytochrome *c* at 550 nm. We then subtracted the absorbance of oxidized cytochrome *c* from that of reduced cytochrome *c* and plotted a standard curve to obtain an extinction coefficient for reduced–oxidized cytochrome *c* at 550 nm of 6.5 mM^−1^. Given the cuvette path length (1 cm), this corresponds to an extinction coefficient of 6.5 mM^−1^ cm^−1^.

NADH’s extinction coefficient at 340 nm was experimentally determined measuring the *A*_340 nm_ of reduced NADH at different concentrations in 20 mM HEPES, pH 7.4, 50 mM NaCl and 10% glycerol (v/v). Measurements of standard curve were done in three replicates with five NADH concentrations over a 10–100 μM range, averaged and background corrected. The standard deviation was used the calculate the error. We determined an extinction coefficient for reduced NADH at 340 nm of 5.4 mM^−1^. Given the cuvette path length (1 cm), this corresponds to an extinction coefficient of 5.4 mM^−1^ cm^−1^.

We calculated the path length of 200 μl of our reaction buffer (20 mM HEPES, pH 7.4, 50 mM NaCl and 10% glycerol (v/v)) in 96-well plates using the Spectramax M2 spectrophotometer’s PathCheck function per the manufacturer’s instructions. Briefly, the PathCheck function was used to determine the path length of 200 μl of our buffer in a 96-well plate, normalized by a PathCheck reference reading of 1 ml of buffer in a 1 cm cuvette, both at 550 nm. Another *A*_550_ reading was done for the 96-well plate without PathCheck. The plate path length was calculated as *A*_550 nm (no PathCheck)_/*A*_550 nm (with PathCheck)_. Measurements of path length were done in three replicates, and the standard deviation was used to calculate the error. We determined the path length of 200 μl of our reaction buffer to be 0.531 cm.

After determining the above, the coefficients of variance of the activity measurements, extinction coefficients and path length were used to calculate the coefficient of variance of the activity assay. The absolute error was calculated by multiplying the average specific activity and the coefficient of variance of the activity assay.

#### NADH-FeCy assay (CI)

Protein sample was added to 1 ml master mix of reaction buffer (20 mM Tris–HCl, 50 mM NaCl and 1 mM FeCy, pH 7.4) and thoroughly mixed by vortexing. The reaction was initiated by the addition of NADH to a final concentration of 200 µM. The wells were mixed by pipetting and plate stirring for 5 s before recording every 4 s for 3 min. Measurements were done in four replicates.

#### NADH-cytochrome *c* assay (SC I + III_2_)

The reaction master mix consisted of 20 mM HEPES, pH 7.4, 50 mM NaCl, 10% glycerol (v/v), 50 U ml^−1^ superoxide dismutase, 100 µM DQ, 4 μM KCN, 100 µM of the corresponding cyt *c*, and the relevant respiratory inhibitor (1 µM antimycin A and 20 µM piericidin A, both dissolved in DMSO). SC I + III_2_ samples were added to the corresponding mix at 7.6 µg ml^−1^ (5 nM), mixed by tumbling and aliquoted into the 96-well plate to a total volume of 200 µl. Each reaction was initiated by addition of 10 µM NADH and briefly mixed by pipetting before recording every 5 s for 10 min. Measurements were done in three to four replicates.

#### A/D transition (CI)

The reaction master mix consisted of 20 mM HEPES, 50 mM NaCl, 10% glycerol (v/v), 0.1% BSA (w/v), 0.1% CHAPS (w/v), 0.1% digitonin (w/v) and 100 μM DQ, pH 7.4. Washed mitochondrial membranes from *V. radiata* or *S. scrofa* were added to the corresponding mix at 40 μg ml^−1^, mixed by tumbling and aliquoted into the 96-well plate to a total volume of 200 μl. For the deactivated condition for *V. radiata*, the plate was incubated at 37 °C for 20 min, after which 15 μM piericidin A (or equivalent amount of DMSO) and 5 μM deaminoNADH (dNADH; or equivalent amount of buffer) were added to the corresponding wells and mixed by pipetting. Ten seconds after this addition, 2 mM NEM or water was added to the corresponding wells and mixed by pipetting. After NEM addition, the plate was incubated at room temperature (25 °C) for 15 min and covered from light. The reactions were started immediately after by the addition of 100 μM NADH or 100 μM dNADH in corresponding wells and briefly mixed by pipetting before recording every 4 s for 5 min. The as-isolated condition assay used a similar set-up, except that no 20 min incubation at 37 °C was applied. Measurements were done in four replicates. For *S. scrofa* membranes, equivalent conditions were used, except that piericidin A and dNADH were not employed. Measurements were done in 6–12 replicates.

### CryoEM grid preparation and data collection

The sample for grid preparation was a heterogeneous sample, namely pooled, concentrated, buffer-exchanged fractions from the sucrose gradient: ~1.5 mg ml^−1^ protein in 30 mM HEPES, 150 mM potassium acetate, 1 mM EDTA and 0.002% (v/v) PMSF, pH 7.7. Digitonin was added to the sample as a last step at a final concentration of 0.2% (w/v) digitonin. Quantifoil 1.2/1.3 mesh copper grids were glow-discharged for 60 s at 30 mA before sample application. Sample (4 µl) was applied to each grid at 10 °C and 90% humidity and incubated on the grid for 20 s before blotting for 4 s and plunge-freezing into liquid ethane using a Leica EM GP2 plunge freezer.

A total of 21,815 high-quality movies were collected using SerialEM v3.8.5 on a 200 kV Glacios microscope equipped with a Quantum K3 detector, at a nominal magnification of 56,818 (0.44 Å per pixel in super-resolution mode). A dose of 20 electrons Å^−2^ s^−1^ with 3 s exposure was fractionated into 75 frames for each movie.

### CryoEM image processing

Raw super-resolution movies were binned two-fold, resulting in a pixel size of 0.88 Å. These movies were motion-corrected using cryoSPARC’s patch-based motion correction, followed by per-micrograph contrast transfer function estimation using CTFFIND4.1, both implemented in cryoSPARC^56^. Particles were initially picked using cryoSPARC’s manual picker, which was then used to train the Topaz^57^ implementation in cryoSPARC, with three iterations. This was followed by 2D classification, 3D ab initio reconstruction and 3D refinement in cryoSPARC. On the last Topaz iteration, 593,080 particles were extracted, downsampled two-fold (pixel size of 1.76 Å) with 300 pixel^2^ boxes and extensively 2D-classified to yield 278,675 particles corresponding to SC I + III2 and 58,798 particles corresponding to CI alone. These particle sets were then re-extracted without down sampling (pixel size of 0.88 Å) with 600 pixel^2^ boxes and used in several rounds of ab initio multi-model generation in cryoSPARC for classification of the particles. These rounds of ab initio model generation removed additional poor-quality particle images and resulted in six classes of SC I + III_2_ particles (two classes with the ferredoxin bridge and four without) and a single class of CI alone particles).

For each class of particles, an initial homogeneous refinement with C1 symmetry was performed followed by iterative rounds of refinement with per-particle defocus, higher-order aberrations and per-particle scale. Finally, a round of non-uniform refinement^58^ was performed, resulting in the final maps for each class. The aligned, scaled and corrected particles from the most closed bridged SC I + III_2_ class (class 1; 123,461 particles) were used in a series of local refinements with masking around distinct parts of the complex. These local maps were combined into a composite map using the Phenix combine maps tool^59^. All software suites used for data processing and refinement except for cryoSPARC were accessed through the SBGrid consortium^60^.

### Model building and refinement

Models for *V. radiata* CI peripheral arm and proximal pumping (P_P_) module and CIII_2_ were used as templates^9,15^. For CI’s distal pumping (P_d_) module and bridge domains, the *A. thaliana* models^11^ were used as starting models after sequence correction into *V. radiata* homologues. These models were fit into the highest-resolution focused refinement maps for separate atomic model building of CI and CIII_2_ in Coot^61^. Real-space refinement of the model was done in Phenix^59^, and group atomic displacement parameters were refined in reciprocal space. Visualization figures were produced in ChimeraX^62,63^.

### Reporting summary

Further information on research design is available in the Nature Portfolio Reporting Summary linked to this article.

### Supplementary information

Reporting Summary

Supplementary Video 1 3D variability analysis of all *V. radiata*’s bridged SC I + III_2_ particles (component 000). The movie shows the limited movement between the matrix and membrane arms of CI and the flexibility of the CI–CIII_2_ interface within the plane of the membrane.

Supplementary Video 2 3D variability analysis of *V. radiata* all bridged SC I + III_2_ particles (component 001). The movie shows the flexibility of the CI–CIII_2_ interface perpendicular to the plane of the membrane.

### Source data

Source Data Extended Data Fig. 1 Uncropped blue-native gels shown in Extended Data Fig. 1b, d, f.

## Figures and Tables

**Extended Data Table 1:**
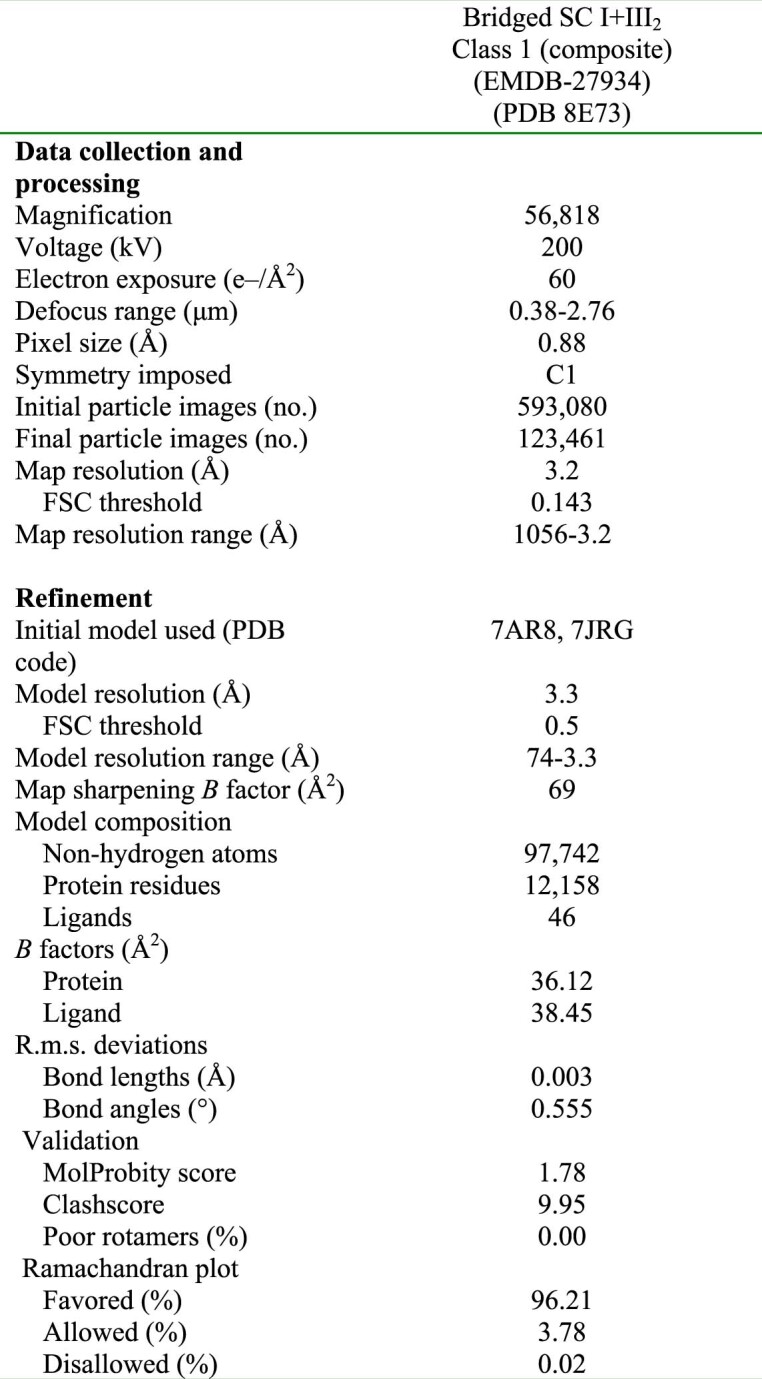
Cryo-EM data collection, refinement and validation statistics for bridged SC I + III_2_ class 1’s composite map and focused refinements

Source data

**Extended Data Table 2:**
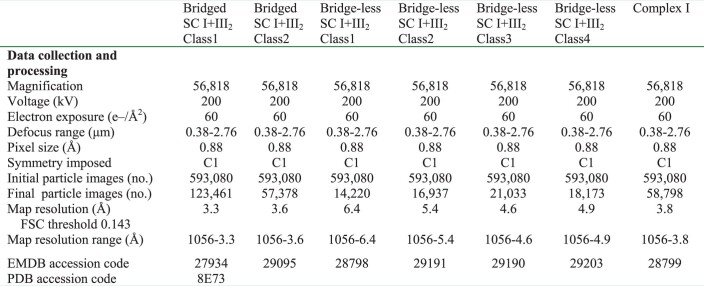
Cryo-EM data collection, refinement and validation statistics for bridged and bridge-less SC I + III_2_ classes, and complex I

**Extended Data Table 3:**
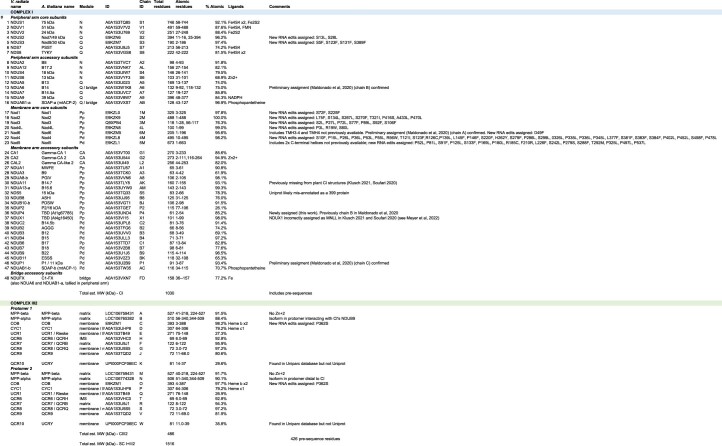
SC I + III_2_ model details by subunit

## Data Availability

Raw cryoEM micrographs used in this study are available on the Electron Microscopy Public Image Archive (EMPIAR) database with accession code EMPAIR-11225. The composite map, focused refinements, and model for V. radiata’s bridged SC I+III_2_ are available on the Electron Microscopy Database (EMDB) and the Protein Data Bank (PDB) with accession codes EMD-27934 for the composite SC I+III_2_ bridged class 1 map and PDB-8E73 for the structural model. Additional maps are available on EMDB with accession codes: EMD-29088 (CI bridge focused); EMD-29089 (CI heel focused); EMD-29090 (CI distal pump domain focused); EMD-29091 (CIII_2_ focused); EMD-29092 (CI N-module focused); EMD-29093 (CIII_2_ proximal MPP domain focused); EMD-29094 (CIII2 distal MPP domain focused); EMD-29095 (SC I+III_2_ bridged class 2); EMDB-28798 (bridge-less SC I+III_2_ classes 1); EMDB-29191 (bridge-less SC I+III_2_ classes 2); EMDB-29190 (bridge-less SC I+III_2_ classes 3); EMDB-29203 (bridge-less SC I+III_2_ classes 4); and EMDB-28799 (CI alone). Source data are provided with this paper.
