# Author Correction: Plant-specific features of respiratory supercomplex I + III_2_ from *Vigna radiata*

**DOI:** 10.1038/s41477-023-01373-5

**Published:** 2023-02-20

**Authors:** M. Maldonado, Z. Fan, K. M. Abe, J. A. Letts

**Affiliations:** 1 Department of Molecular and Cellular Biology, University of California, Davis, CA USA; 2 Present Address: Department of Plant Biology, University of California, Davis, CA USA

**Keywords:** Cryoelectron microscopy, Biochemical assays, Plant sciences, Plant molecular biology

Correction to: *Nature Plants* 10.1038/s41477-022-01306-8. Published online 29 December 2022.

In the version of this article initially published, a concluding statement was missing, which explained that the paper was co-submitted with the article from reference 53 on the *Arabidopsis* supercomplex (Klusch, N. et al. *Nat. Plants* 10.1038/s41477-022-01308-6 (2023)). The statement can now be found under the Discussion section. The error has been corrected in the HTML and PDF versions of the article.

