# Supplementary figures and images for: Plant-specific features of respiratory supercomplex I + III2 from Vigna radiata
Source: Nat Plants. 2022 Dec 29;9(1):157–68. doi: 10.1038/s41477-022-01306-8 (PMC9873571; doi:10.1038/s41477-022-01306-8)

Source Data Extended Data Figure 1.

Panel b:

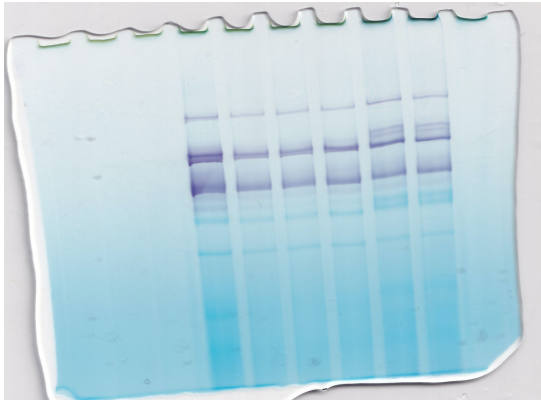

Panel d:

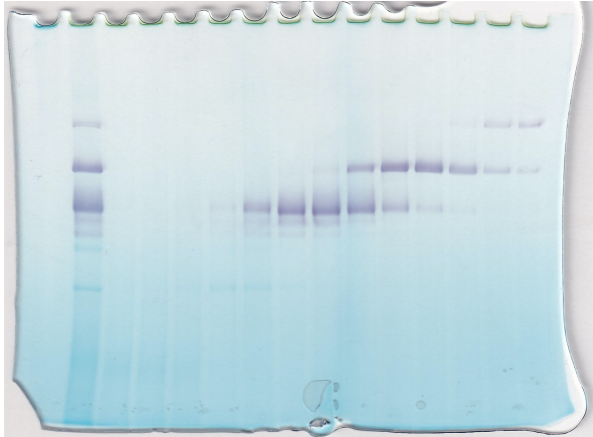

Panel f:

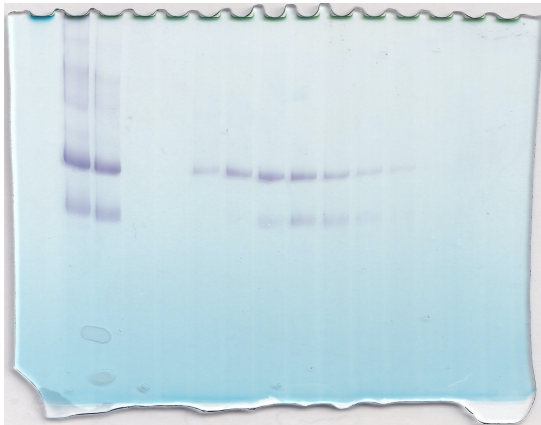

Supplement: Supplementary file 4 — Uncropped blue-native gels shown in Extended Data Fig. 1b, d, f. [file 41477_2022_1306_MOESM4_ESM.pdf]
